# Bovine Pericardial Patch Augmentation of One Insufficient Aortic Valve Cusp with Twenty-three-year Positive Clinical Follow-up Independent of the Patch Degeneration

**DOI:** 10.21470/1678-9741-2017-0010

**Published:** 2017

**Authors:** Paulo Roberto Barbosa Evora, Lívia Arcêncio, Patrícia Martinez Evora, Antônio Carlos Menardi, Fernando Chahud

**Affiliations:** 1 Department of Surgery and Anatomy, Faculdade de Medicina de Ribeirão Preto da Universidade de São Paulo (FMRP-USP), Ribeirão Preto, SP, Brazil.; 2 Department of Animal Pathology, Faculdade de Veterinária da Universidade Estadual Paulista (UNESP), Campus of Jaboticabal, Jaboticabal, SP, Brazil.; 3 Department of Pathology, Faculdade de Medicina de Ribeirão Preto da Universidade de São Paulo (FMRP-USP), Ribeirão Preto, SP, Brazil.

**Keywords:** Aortic valve/surgery, Aortic valve insufficiency, Heart valve diseases, Cardiovascular surgical procedures, Pericardium

## Abstract

Scientific progress shall ultimately boost the current acceptance level for conservative aortic valve surgery. The present text aimed to report the 23-year long-term follow-up of one patient operated with bovine pericardium cusp extension. Growing confidence in the efficacy of the operation will allow a more expeditious indication for surgical treatment, as is already the case in mitral valve repair. This change of attitude will certainly make it possible for patients to be sent for operation in mild aortic valve regurgitation. The present report reinforces the concept and highlights the impression that the aortic valvoplasty, independent of the progressive bovine pericardium degeneration, may positively change the natural history of the aortic valve insufficiency.

**Table:** 

Abbreviations, acronyms & symbols	
NYHA	= New York Heart Association

## INTRODUCTION

Aortic valve repair, one of the first procedures performed in the history of heart surgery, was somewhat ignored compared to valve replacement by artificial prosthesis, which became very popular in the sixties. Difficulties related to myocardial protection in association with the relative paucity of intraoperative and postoperative technical resources available, as well as the apparent simplicity of valve replacement procedures, were responsible for this initial indifference towards aortic valve reconstructive surgery.

From August 1988 to June 1990, six patients were operated on by bovine pericardium cusp augmentation^[1,2]^. Mild aortic valve regurgitation was a universal finding in early postoperative period, which gradually worsened over the first years. The present article aimed to report the anecdotal 23-year long-term follow-up of one case, among the cohort group, suggesting a positively natural history of aortic valve insufficiency independent of the progressive patch degeneration.

## CASE PRESENTATION

This case report refers to a 57-year-old female patient, with rheumatic antecedent related in May 1994, presenting progressive dyspnea, decubitus dyspnea and paroxysmal nocturnal dyspnea and New York Heart Association (NYHA) functional class IV. Pulmonary auscultation was normal; blood pressure was 120/40 mmHg and heart rate was 72 bpm. The cardiac auscultation revealed intense diastolic murmur in the aortic focus.

Pressures (mmHg) measured by cardiac catheterization in April 1992 were: aorta (155/85 average 110); pulmonary artery (27/12 average 18); pulmonary capillary wedge pressure (15/8 average 12); left ventricular (135/0 average 12); right atrium (mean 5) and right ventricle (25/3 average 8).

The preoperative Doppler echocardiography showed a normal left ventricular function, hypertrophy, moderate stenosis, severe aortic valve regurgitation and a discrete mitral valve insufficiency. The aortic valve presented three thickened leaflets with opening and reduced mobility (gradient: 63 mmHg/33 mmHg, estimated area 1.3 cm²). Also, chest aorta holodiastolic reflux was observed, with “slope” of 412 cm/s² associated with downward abdominal aorta protodiastolic reflux.

### Surgical Technique

**Fig. 1:**
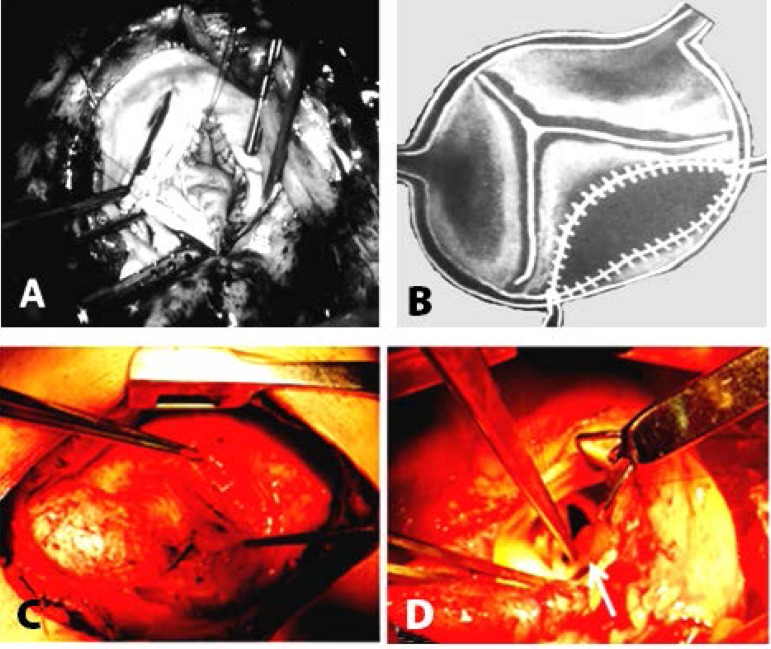
A and B - Schematic representation of the advanced of noncoronary cusp showing the semilunar pericardium shape; C - Aorta aspect; D - Intraoperative aspect of the calcified right coronary aortic cusp amplification with bovine pericardium including the base implantation of the cusp free edge.

After cardioplegia administration directly into the coronary ostium, a temporary fine suture was then applied to temporarily bring together the nodule of Arantius. A partial detachment of the retracted cusp was carried out, the bovine pericardium patch was sutured at the commissural level, followed by the commissures resuspension. The bovine pericardium patch must be tailored to an oval shape with its major axis equal to the cusp base (Figure 1).

After surgery, the patient remained asymptomatic for about six months, only with the use of hydrochlorothiazide. After this period, she started to show symptoms on effort (NYHA class II). Echocardiographic monitoring showed progressive degeneration of valvuloplasty. In 1998, in the use of digoxin and captopril, an exercise stress test revealed normal physical fitness. In 2001, reoperation was proposed and was refused. The situation remained unchanged until 2016 when the patient was reoperated for implantation of a biological valve prosthesis.

**Fig. 2:**
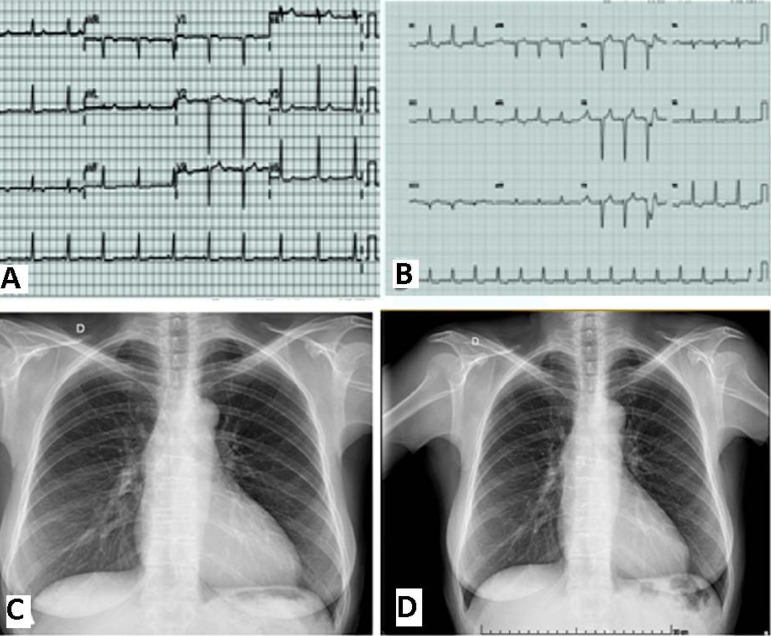
Quite similar electrocardiogram records A - Year 2009; B - Year 2016; Quite similar chest X-ray. C - Year 2015; D - Year 2016.

**Fig. 3:**
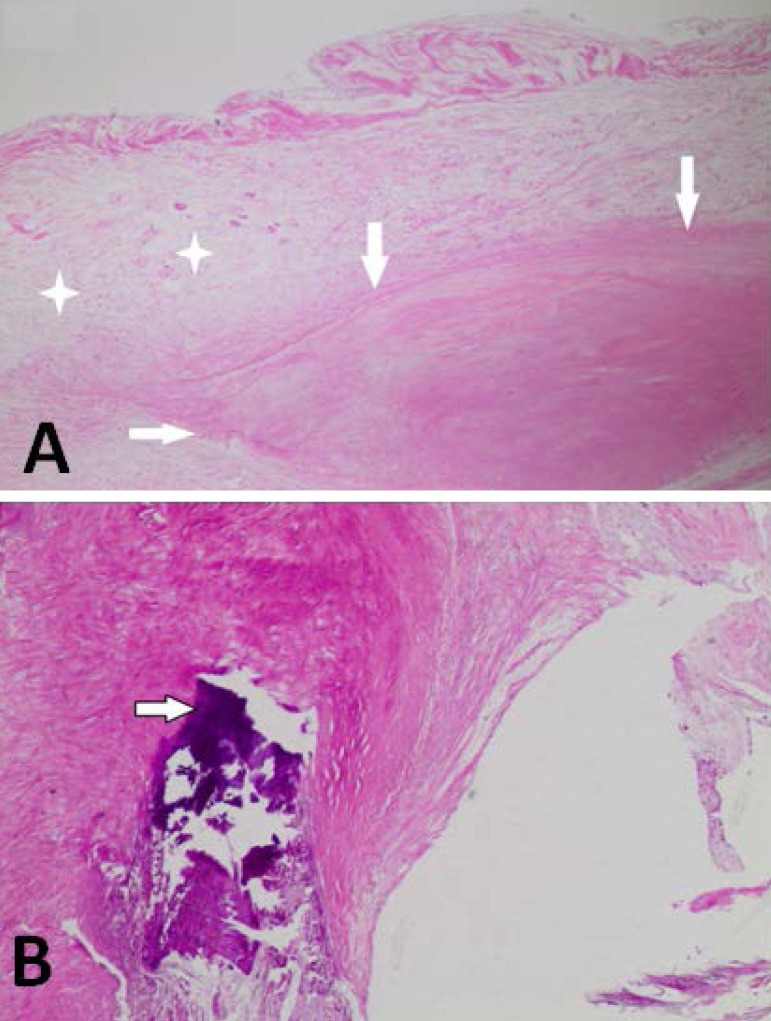
A - The excised valve showed areas of dense fibrous tissue (arrows) e focal myxoid degeneration (stars); B - Focal areas of calcification (arrow) were also evident (H&E, 40x).

The case report is illustrated by electrocardiogram and chest X-ray (Figure 2), echocardiogram data (Table 1), and histology of the excised valve (Figure 3).

**Table 1:** Echocardiogram data.

	2012	2015	2016	Reference values
Aorta (root)	30	31	32	(20 – 37 mm)
Transverse aorta diameter	26	27	26	(up to 32 mm)
Left atrium	47	45	46	(20 – 40 mm)
Left ventricle end diastolic diameter	64	66	66	(35 – 36 mm)
Left ventricle end systolic diameter	39	43	44	(25 – 40 mm)
Ejection fraction	68	63	58	(> 50%)
Left ventricle mass index	187.43	162.52	232.86	(up to 110 g/m^2^)

## DISCUSSION

Until 1988, a literature review, so widely as possible, did not reveal any similar description to the proposed aortic valve reconstruction technique with bovine pericardium, thus this operation should surgically be an original contribution. The surgery was an adaptation of the Sauvage & Wood^[3]^ proposed for treating the mitral insufficiency by advancing the posterior leaflet with a semilunar autologous pericardium patch^[3]^. The main difference of this reconstructive technique, particularly with the technique used by al Fagih et al.^[4]^, lies in the fact that it is carried out from their annular detachment and not from its free edge. This fact may decrease the mechanical fatigue of bovine pericardium and be associated with lower valvular dysfunction incidence in the postoperative period.

Some considerations on the acquired experience with this technique can be summarized, so far: 1) the technique is reproducible with good immediate results; 2) one or more cusps can be enlarged, and one should select the most damaged one(s); 3) by adding other reconstructive procedures in association to this technique, the results should improve; 4) the long-term behavior of the native cusp tissue regarding its further fibrotic degeneration is also worrisome; 5) as is the case for the mitral valve, it seems that the bovine pericardium patch outlives the native cusp, and; 7) long-term clinical evolution must be awaited for a more definitive appreciation of the results, although, so far, clinical results seem similar to the ones afforded by aortic valve bioprosthetic replacements^[2]^.

Preoperative and intraoperative evaluations are essential for proper selection of the techniques to be applied in each particular case. The surgeon should aim at readjusting the positioning and shape of the cusps so that a perfect valvular coaptation is regained during ventricular diastole, thus correcting the regurgitant lesion. After completing surgical correction, transesophageal echocardiography is of utmost importance in evaluating the immediate functional result at the end of cardiopulmonary bypass and after the normal heart function is re-established.

## CONCLUSION

In conclusion, scientific progress shall ultimately boost the current acceptance level for conservative aortic valve surgery. Growing confidence in the efficacy of the operation will allow a more expeditious indication for surgical treatment, as is already now the case in mitral valve repair. This change of attitude will certainly make it possible for patients to be sent for operation in mild aortic valve regurgitation which, our experience shows, gradually worsens over the first years. Otherwise, based on our previous experience, the patients continue clinically well, with better ventricular function and lesser degrees of structural valvar degeneration. The present report reinforces this concept and highlights the impression that the aortic valvoplasty, independent of the progressive bovine pericardium degeneration, changed positively the natural history of the aortic valve insufficiency.

**Table:** 

Authors’ roles & responsibilities	
PRBE	Study design and manuscript writing; final manuscript approval
LA	Study design and manuscript writing; final manuscript approval
PME	Study design and manuscript writing; final manuscript approval
ACM	Study design and manuscript writing; final manuscript approval
FC	Critical review of the manuscript; final manuscript approval
